# A state-of-the-art review of functional magnetic resonance imaging technique integrated with advanced statistical modeling and machine learning for primary headache diagnosis

**DOI:** 10.3389/fnhum.2023.1256415

**Published:** 2023-09-01

**Authors:** Ming-Lin Li, Fei Zhang, Yi-Yang Chen, Han-Yong Luo, Zi-Wei Quan, Yi-Fei Wang, Le-Tian Huang, Jia-He Wang

**Affiliations:** ^1^Department of Family Medicine, Shengjing Hospital of China Medical University, Shenyang, Liaoning, China; ^2^Department of Family Medicine, Liaoning Health Industry Group Fukuang General Hospital, Fushun, Liaoning, China; ^3^Department of Oncology, Shengjing Hospital of China Medical University, Shenyang, Liaoning, China

**Keywords:** functional magnetic resonance imaging, pathophysiology, machine learning, statistical modeling, primary headaches, diagnosis

## Abstract

Primary headache is a very common and burdensome functional headache worldwide, which can be classified as migraine, tension-type headache (TTH), trigeminal autonomic cephalalgia (TAC), and other primary headaches. Managing and treating these different categories require distinct approaches, and accurate diagnosis is crucial. Functional magnetic resonance imaging (fMRI) has become a research hotspot to explore primary headache. By examining the interrelationships between activated brain regions and improving temporal and spatial resolution, fMRI can distinguish between primary headaches and their subtypes. Currently the most commonly used is the cortical brain mapping technique, which is based on blood oxygen level-dependent functional magnetic resonance imaging (BOLD-fMRI). This review sheds light on the state-of-the-art advancements in data analysis based on fMRI technology for primary headaches along with their subtypes. It encompasses not only the conventional analysis methodologies employed to unravel pathophysiological mechanisms, but also deep-learning approaches that integrate these techniques with advanced statistical modeling and machine learning. The aim is to highlight cutting-edge fMRI technologies and provide new insights into the diagnosis of primary headaches.

## 1. Introduction

### 1.1. Overview of primary headaches

Primary headaches, which do not stem from any underlying disease or secondary cause, are classified into four main categories according to the International Classification of Headache Disorders (ICHD-3) (Arnold, 2018) migraine, tension-type headache (TTH), trigeminal autonomic cephalalgia (TAC), and other primary headaches. Recent research shows that worldwide, around 52.0% (95% CI: 48.9–55.4) of people are affected by active headache disorder (Stovner et al., 2022), while findings from the 2019 Global Burden of Disease (GBD) study report a prevalence of 35.0% (32.3–37.7) for primary headaches (Steiner et al., 2020). Furthermore, a comprehensive review of the prevalence of primary headaches in child and adolescent was shown to be 62% (53–70) (Onofri et al., 2023). Primary headaches, chiefly TTH and migraines, have prevalences of 26.0% (22.7–29.5) and 14.0% (12.9–15.2), respectively (Stovner et al., 2022). TACs alongside other primary headaches are relatively rare, with incidence rates of about 0.05–0.1% in the population (Eller and Goadsby, 2016; Kaniecki and Levin, 2019; Kopel and Gottschalk, 2022; Nägel and Kraya, 2022). Headache disorders impose a considerable burden and are highly prevalent across the globe. Globally, headache disorders are ranked by the GBD as the second major source of disability-adjusted life years (DALYs) (Saylor and Steiner, 2018). The growing burden on individuals, society, and the economy makes it essential to adopt different management and treatment approaches based on headache classifications (Weeks, 2022). However, a prerequisite for this is an accurate diagnosis.

Primary headaches currently lack clear radiological and physiological biomarkers, so their diagnostic criteria are still based on symptomatology (Levin, 2022). However, clinical features are often similar among different categories and complex situations, such as varying severity levels or comorbidities, may make it challenging to distinguish diagnoses based solely on these features. As research on the pathogenesis of primary headaches deepens, an increasing number of imaging, genetic, and neurochemical markers are being discovered. Functional magnetic resonance imaging (fMRI) technology, which has better temporal and spatial resolution and which can be used to explore the underlying pathophysiology of primary headaches, has become a hot research topic because it can reliably and objectively differentiate different categories of primary headache patients as a diagnostic tool (Glover, 2011; Naegel and Obermann, 2021).

### 1.2. Introduction to fMRI technology

Utilizing ultra-fast imaging sequences, fMRI is a technique specifically designed to diagnose diseases by identifying functional changes before they lead to pathological alterations detectable by traditional MRI methods. Broadly, current fMRI methods mainly include diffusion-weighted imaging (DWI), diffusion tensor imaging (DTI), perfusion-weighted imaging (PWI), magnetic resonance spectroscopy (MRS), and brain cortical function localization techniques (primarily utilizing blood oxygen level-dependent (BOLD) effects). Among these, brain cortical function localization is widely applied, so fMRI typically refers to BOLD-based functional magnetic resonance imaging (BOLD-fMRI, hereinafter known simply as fMRI). Studies have shown that even at rest, brain energy consumption accounts for around 20% of the body’s total energy expenditure, with 60–80% of that energy used for communication between neurons and their supporting cells (Raichle and Mintun, 2006). Brain neuronal activity correlates with changes in blood oxygen levels, and variations in the ratio between highly paramagnetic deoxyhemoglobin and diamagnetic oxyhemoglobin cause local field inhomogeneity, leading to alterations in T2-weighted images. This enables the imaging of brain function activity, brain tissue metabolism, and alterations in local brain blood circulation, which is the main theory behind BOLD-fMRI (Ogawa et al., 1990; Sun et al., 2021).

Widely utilized to investigate the functions and interactions of particular brain regions, fMRI can be categorized into two types: task-state fMRI (ts-fMRI) and resting-state fMRI (rs-fMRI). These classifications depend on whether the patient must perform specific tasks or receive predetermined external stimuli during the imaging process. Due to the existence of spontaneous low-frequency BOLD fluctuations in any brain region during rest, as well as the fact that induced activity increases neuronal metabolism by less than 5%, rs-fMRI has gained popularity in clinical research because it doesn’t necessitate participants performing multiple tasks (Smitha et al., 2017). Nonetheless, task design remains crucial for eliciting meaningful brain activation and associated functional connectivity patterns (Jiang et al., 2020; Zhao et al., 2023). FMRI imaging technology requires data acquisition and reconstruction, pre-processing (temporal calibration, head movement correction, registration and normalization, spatial smoothing), and data analysis (localization of brain activity, connection strength analysis, prediction). When analyzing brain activity, attention must be given to both local activity and functional connectivity (FC) between various regions of the brain, employing two main rs-fMRI analysis methods: functional segregation and functional integration (Biswal et al., 1995; Fox and Raichle, 2007; Lv et al., 2018; Schramm et al., 2023; Supplementary Table 1). Functional segregation primarily concentrates on the distinct functions of various brain regions, with main analysis techniques including amplitude of low-frequency fluctuations (ALFF) and regional homogeneity (ReHo). Functional integration, on the other hand, incorporates brain activity into interconnected networks, mainly focusing on the interaction between different brain regions. Common methods include functional connectivity density (FCD) analysis, seed-based FC analysis, independent component analysis (ICA), and graph theory-based brain network analysis. Dynamic associations and connections between various brain regions constitute the brain’s functional networks, such as the default mode network (DMN), salience network (SN), and frontoparietal network/central executive network (FPN/CEN) which are most relevant to pain detected by fMRI (Fox et al., 2005; van den Heuvel and Hulshoff Pol, 2010; Naegel and Obermann, 2021). Different from the data-driven rs-fMRI, ts-fMRI is model-driven, requiring meticulous experimental design to clarify baselines, number of baseline and task alternations, start and duration times, etc. Mainstream analysis methods include generalized linear regression models and hemodynamic response functions (Chen and Glover, 2015).

As multi-center big data research deepens, the study of brain abnormalities is transitioning from group-based research to individual studies, providing solutions for precise clinical diagnosis and targeted therapy of brain disorders. Technological advancements have facilitated high-quality fMRI with higher spatiotemporal resolutions, yielding richer local anatomical information and more accurate functional signals, thereby promoting finer-grained brain function research. The ubiquitous access to fMRI technology, its non-intrusive nature, relative affordability, and commendable spatiotemporal resolution have progressively strengthened its indispensable status within the realm of functional neuroscience, such as brain cognition, aging, and major brain diseases, among others (Hranilovich et al., 2023).

## 2. The role of fMRI technology in diagnosing primary headaches

### 2.1. Background

With the continuous development of medical-engineering integration, computer vision tasks, including image classification, object detection, image segmentation, object tracking, pose estimation, and image generation, have been widely used in many fields (Zhang J. et al., 2021; Li et al., 2022a), such as automatic supervision and differentiation of diseases such as the digestive system (Barmpoutis et al., 2022; Chen H. et al., 2022), nervous system (Qureshi et al., 2019; Hu et al., 2023), and reproductive system (Liu et al., 2022; Fan et al., 2023) and so on. However, to date, no medical imaging technology has been able to definitively diagnose or differentiate primary headache disorders. Although conventional neuroimaging techniques are widely used in clinical for headache patients, they primarily serve as an exclusionary diagnosis in most cases (Tedeschi et al., 2012). Consequently, an increasing number of studies are investigating novel imaging diagnostic approaches. FMRI has increasingly been employed to explore the pathophysiological underpinnings of primary headaches, contributing to a more comprehensive understanding of brain activity networks and identifying potential intervention targets. Therefore, fMRI offers a promising avenue for improving the diagnosis of primary headaches.

Although still in its nascent stages in the investigation of primary migraines, recent studies suggest that beyond the fundamental pathophysiological exploration using fMRI, the application of advanced statistical modeling and machine learning techniques offers new possibilities for the differentiation and diagnosis of primary headaches. Common machine learning algorithm categories (Raschka, 2018) include supervised learning, semi-supervised learning, unsupervised learning, and reinforcement learning. Fundamental models encompass Linear Regression, Logistic Regression, Decision Trees, Random Forests, Support Vector Machines (SVM), K-Nearest Neighbors, Neural Networks (NNs), and Naive Bayes, each with its respective applicability and strengths and weaknesses (Supplementary Table 2). Notably, the rapidly evolving and currently prominent Deep Learning is an extension of NNs (LeCun et al., 2015). By autonomously learning intricate feature representations and representation learning, it excels in tasks such as medical image analysis, disease classification, and prediction.

Within deep learning, there are several commonly used neural network architectures. The Multilayer Perceptron (MLP) (Rumelhart et al., 1986), a foundational feed-forward neural network, comprises multiple fully connected layers and is employed for tasks such as classification, regression, and clustering. The Convolutional Neural Network (CNN) (Szegedy et al., 2015; Messaoudi et al., 2023), characterized by its convolutional and pooling layers, has seen notable enhancements through modules like the Inception and the ResNet residual blocks. CNNs, primarily tailored for image processing, have achieved significant breakthroughs in computer vision tasks. Recurrent Neural Networks (RNNs) (Hochreiter and Schmidhuber, 1997; Cho et al., 2014), which introduce a consideration for sequences data atop the CNN framework, possess a cyclic architecture and are predominantly utilized for language modeling and video data processing. Long Short-Term Memory (LSTM) and Gated Recurrent Unit (GRU) are more advanced modules common to RNNs. Autoencoder (Vincent et al., 2010) learns efficient data representations through the reconstruction of training data, including encoders and decoders, which can be used for tasks such as data dimensionality reduction, feature extraction, and model generation. Common improved models include Sparse Autoencoder and Variational Autoencoder. The Attention Mechanism (Vaswani et al., 2017), which uses attention mechanisms to process sequence and image data, is widely used in tasks such as machine translation, image description generation and speech recognition, among which the classic improvement module is the Transformer module.

These neural architectures have found broad applications; for instance, a CNN model, integrating convolutional and fully connected layers, achieved a diagnostic accuracy of 92.87% in distinguishing Alzheimer’s Disease (AD) from healthy controls (HC) (Li et al., 2014). Similarly, the Inception-based GoogleNet reported an impressive accuracy of 98.74% in differentiating AD from HC (Sarraf and Tofighi, 2016). Furthermore, a proposed 3D-CNN-LSTM classification model has demonstrated an accuracy of 96.4% in discerning varying degrees of cognitive impairment (Noh et al., 2023). These deep learning-based frameworks are of great significance for the classification of brain diseases in clinical trials and large-scale studies, and by combining multimodal fMRI data and clinical information, not only can they reveal the functional connections and functional networks between brain regions, machine learning models are also able to learn the pathological features of complex neural activities from large-scale data, and through continuous improvement, the classification accuracy of specific diseases can be further improved.

**FIGURE 1:**
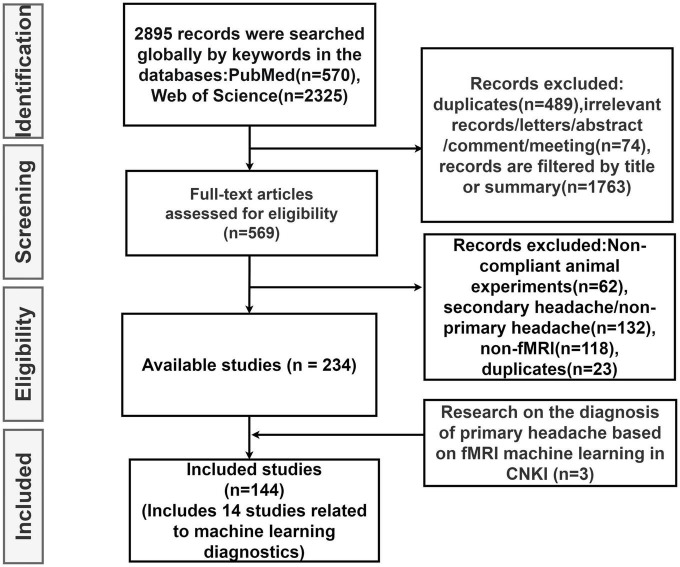
Literature search flow chart.

**FIGURE 2:**
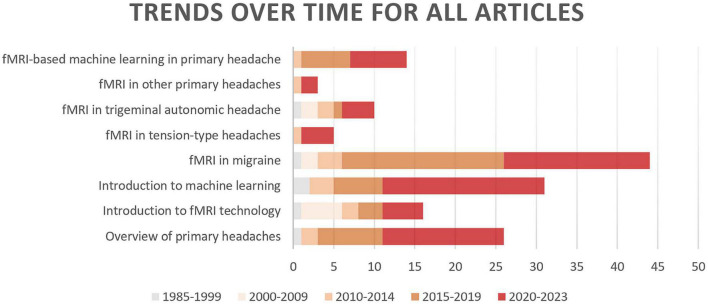
Trends over time for all articles included in this review.

In this review, we first explore the pathophysiology of various subtypes of primary headache based on BOLD-fMRI imaging technology, and summarize the functional separation and functional integration between brain regions, which may provide basic data for further machine learning. Then, the research progress of using advanced statistical modeling and machine learning methods to distinguish or diagnose primary headache based on fMRI data is mainly summarized. As machine learning is not widely used in the field of primary headache, in order to summarize machine learning-related research more comprehensively, we conducted an exhaustive search (Figure 1) in PubMed, Web of Science and CNKI, using keywords related to primary headache and its subtypes, functional magnetic resonance imaging, statistical modeling, machine learning, and diagnosis (including classification or differentiation). The titles and abstracts of each study were independently evaluated by the authors, reaching a consensus after detailed scrutiny and discussion in case of conflicts. Reference lists from each study were manually assessed to avoid the omission of relevant research. Upon screening studies based on titles and abstracts, the authors independently reviewed the full texts of the remaining articles. After examining the full texts, research findings relevant to the central theme of this paper were incorporated for further analysis. Figure 2 summarizes trends over time for all articles included in this review.

**FIGURE 3:**
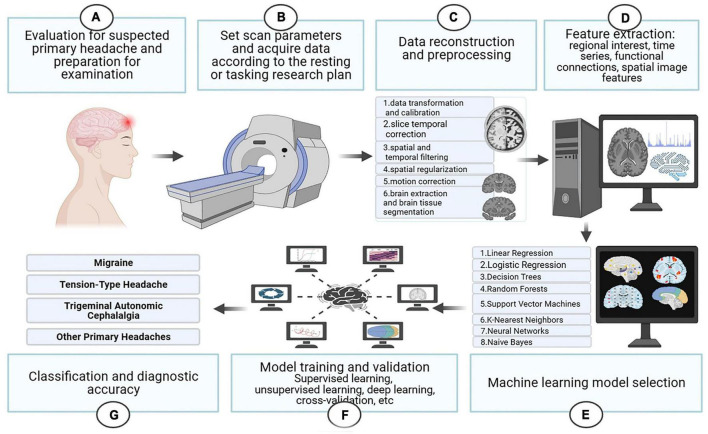
The basic process of functional magnetic resonance imaging combined with machine learning for the diagnosis of primary headache.

The following we discuss the subtypes of primary headache separately, the first part of each subtype is a pathophysiological discussion, which we summarize according to different brain regions in the order of different analysis methods. The second part is summarized according to the different headache categories, in order of diagnostic accuracy from highest to lowest and the specific characteristics of the included studies are summarized in Supplementary Table 3 and the basic process of functional magnetic resonance imaging combined with machine learning for the diagnosis of primary headache is shown in Figure 3.

### 2.2. Diagnosis of migraine based on fMRI technology

#### 2.2.1. Exploring the pathophysiology of migraine diagnosis through fMRI technology

**FIGURE 4:**
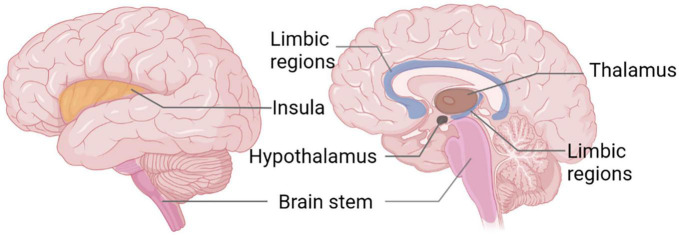
Functional magnetic resonance imaging (FMRI) studies reveal recurrent brain areas in migraine patients.

Migraine is a genetically determined disorder of cerebral excitability that manifests as an intermittent and recurrent condition, leading to activation and sensitization of the trigeminovascular pain pathway (Pietrobon and Moskowitz, 2013). Clinically, it is predominantly characterized by episodic moderate-to-severe, pulsating, unilateral headaches. Furthermore, it is frequently accompanied by symptoms of nausea and vomiting. Light and sound stimuli, as well as daily activities, can exacerbate the headache. Although the prevalence of migraines is not as high as TTH, migraine patients frequently exhibit cognitive and psychological abnormalities, with more severe symptoms, more complex pathogenesis, and greater treatment demands (Gu et al., 2022; Vicente et al., 2023). This is why current research on migraines is more extensive and in-depth. While often linked to a variety of internal and external influencers, the neurovascular processes that contribute to the onset of migraines, a fundamental neurological disorder, continue to necessitate more in-depth understanding and analysis (Russo et al., 2019a). Evidence has demonstrated that hypothalamic activation can be detected within 48 h prior to the onset of a headache, which has been interpreted as a potential marker for the prodromal phase (Schulte et al., 2020). The brainstem was activated continuously while the migraine occurred, and the activation of the midbrain, hypothalamus and cortex were also shown during this period (Weiller et al., 1995; Bahra et al., 2001; Denuelle et al., 2007). Rs-fMRI research has uncovered variations in the functionality of certain brain areas in individuals suffering from migraines during interictal and ictal phases, including recurrent involvement of the insula, brainstem, limbic system, hypothalamus, and thalamus (Figure 4) and the most prominent changes within FC networks include salience, frontoparietal, executive, and sensorimotor networks (Damoiseaux et al., 2006; Raichle, 2015); these alterations also correlate with migraine duration, attack frequency, and pain intensity (Schwedt and Chong, 2015; Coppola et al., 2016; Andreou and Edvinsson, 2019; Huang et al., 2019; Torres-Ferrús et al., 2020; Ashina et al., 2021).

**FIGURE 5:**
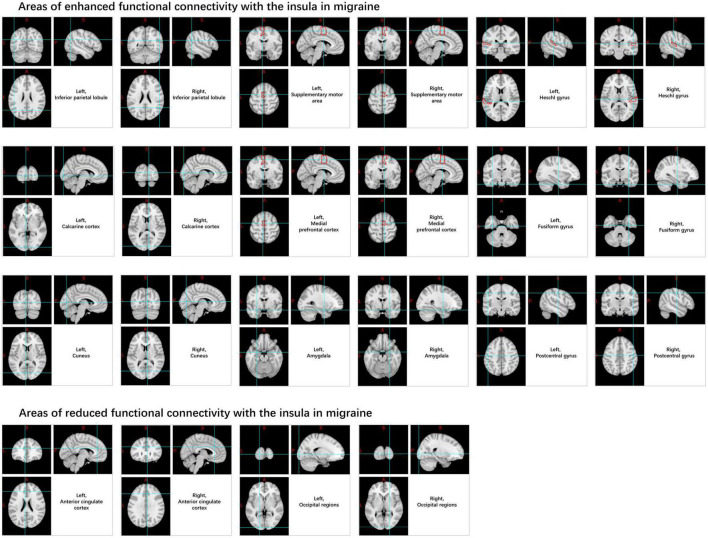
Changes in functional connectivity in the insular in migraine patients.

Compared to the HC, migraine patients exhibit increased ALFF values and BOLD signal variability in the insular cortex under rs-fMRI. The resting-state FC (rs-FC) between the insula and medial prefrontal cortex, inferior parietal lobule, Heschl’s gyrus, pons, calcarine cortex, amygdala, cuneus, supplementary motor area, central posterior gyrus, temporal lobe, fusiform gyrus, and cerebellum is enhanced (Yu Z. B. et al., 2017; Coppola et al., 2018; Ke et al., 2020). In contrast, connectivity decreases in the anterior cingulate cortex and occipital regions (Niddam et al., 2016; Zhang et al., 2020; Figure 5).

**FIGURE 6:**
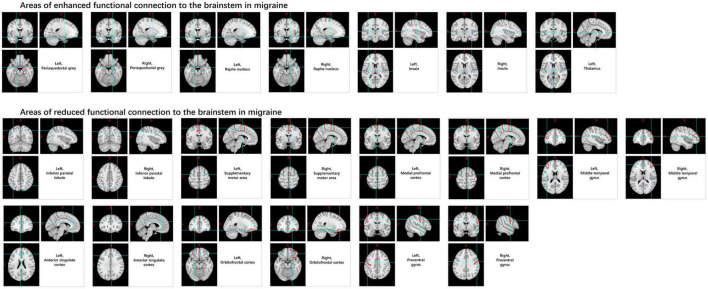
Changes in functional connectivity in the brainstem in migraine patients.

Numerous brainstem nuclei play roles in different types of migraines, including the red nucleus, substantia nigra, periaqueductal gray (PAG), spinal trigeminal nucleus, and the median raphe nucleus, among others. Among these, the most extensively studied structure is the spinal trigeminal nucleus. Research indicates that compared with HCs, migraine patients show increased variability in BOLD signals, ReHo, and power of sub-low frequency oscillations in the spinal trigeminal nucleus. Compared to HCs, FC is enhanced in migraine patients between the brainstem and the periaqueductal gray matter, median raphe nucleus, insula, and thalamus, and it decreases between the medial prefrontal cortex, middle temporal gyrus, orbital cortex, anterior cingulate cortex, brainstem, the inferior parietal lobule, pre-central gyrus, supplementary motor area, and the spinal trigeminal nucleus. However, contradictory results have been shown in the hypothalamus, indicating a need for further investigation (Lee et al., 2017; Schramm et al., 2023; Figure 6).

Moreover, compared to HC, migraineurs exhibit increased rs-FC between the PAG surrounding the midbrain and the median raphe nucleus, ventromedial medulla oblongata, adjacent gray matter around the aqueduct, hypothalamus, and thalamus. Similarly, increased ReHo and sub-low frequency oscillation power in the PAG and hypothalamus were observed in task-based fMRI before migraine attacks (Meylakh et al., 2018). In contrast, decreased rs-FC was found between the medial prefrontal cortex, orbitofrontal cortex, and anterior cingulate cortex (Chen et al., 2017). The duration and intensity of migraine pain have been shown to be positively correlated with rs-FC of the PAG and substantia nigra, and with the ALFF values of the bilateral ventrolateral posterior thalamic nuclei and brainstem regions, including the ventromedial medulla and the trigeminal-cervical complex (Huang et al., 2019; Kim Y. E. et al., 2021). Conversely, a negative correlation was found between the fractional ALFF (fALFF) values of the left middle frontal gyrus and migraine pain intensity (Wu et al., 2023).

**FIGURE 7:**
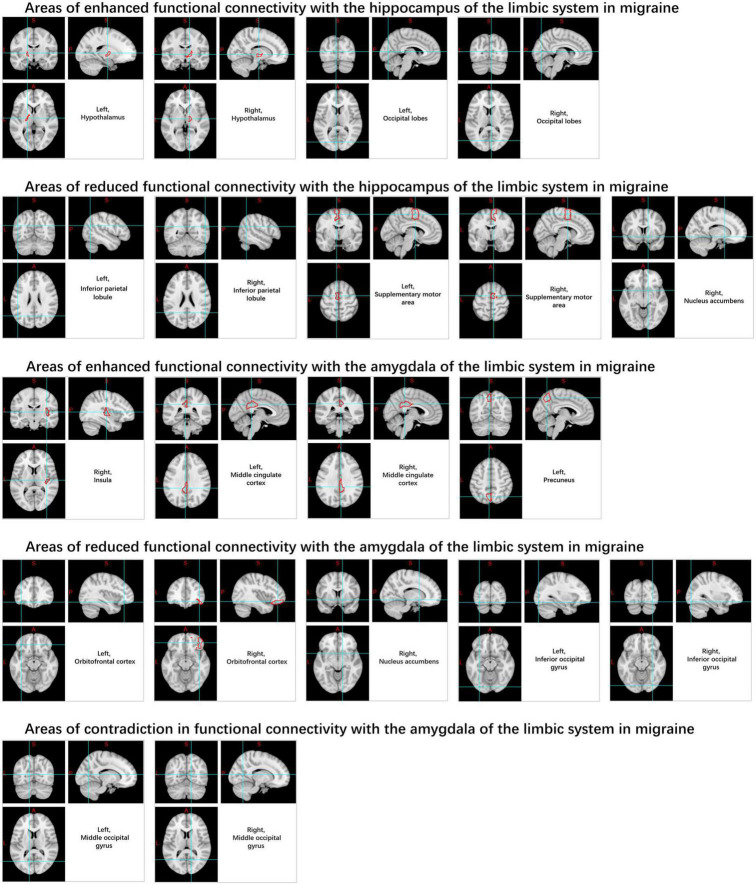
Changes in functional connectivity in the limbic system in migraine patients.

The limbic system (Figure 7) has been identified as being associated with migraines, particularly the amygdala, cingulate cortex, and hippocampus (Wei et al., 2020; Zhang et al., 2020). It was observed that rs-FC increased between the posterior cingulate cortex, middle cingulate cortex, right insula and left anterior cingulate cortex and almond nucleus, while rs-FC decreased between the orbitofrontal cortex and right nucleus. The rs-FC of the hippocampus is increased in the hypothalamus, cerebellum, and occipital lobe regions, and decreased in the right nucleus accumbens, inferior parietal lobule, and supplementary motor area. Migraine sufferers also exhibit increased variability in the rs-BOLD signal within the hippocampus (Lim et al., 2021). However, there is still considerable heterogeneity in the rs-FC of the cingulate cortex in current studies (Yu D. et al., 2017; Zhang et al., 2020; Kim D. J. et al., 2021).

**FIGURE 8:**
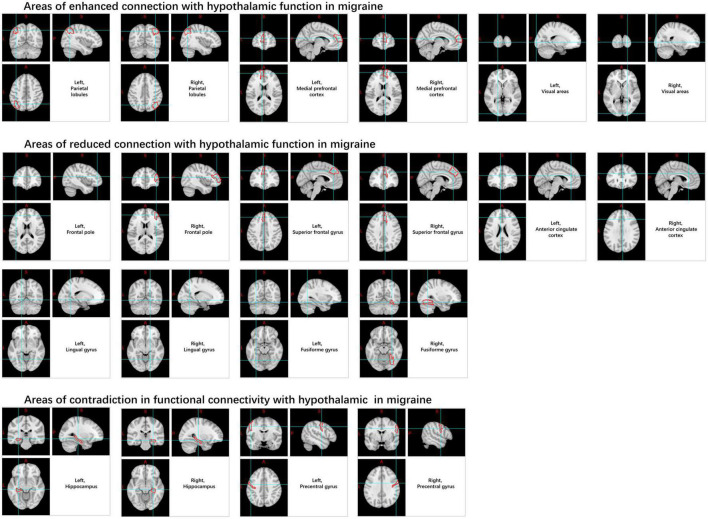
Changes in functional connectivity in the hypothalamus in migraine patients.

Likewise, when compared to HC, there was an observed increase in rs-FC from the hypothalamus to the medial prefrontal cortex, parietal leaflets, visual areas, pre-central gyrus, hippocampus, pons regions, and several other regions in patients with migraines, as well as decreased rs-FC in the hippocampal formation, anterior cingulate cortex, PAG, spinal trigeminal nucleus, rostral ventromedial medulla, pontine area, pre-central gyrus, frontopolar cortex, superior frontal gyrus, fusiform gyrus, and lingual gyrus (Moulton et al., 2014; Coppola et al., 2020; Meylakh et al., 2020; Figure 8).

**FIGURE 9:**
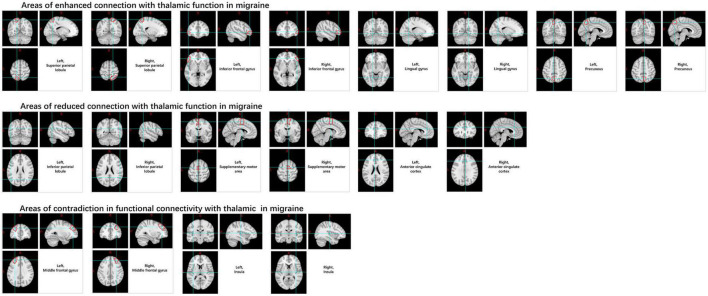
Changes in functional connectivity in the thalamus in migraine patients.

In the thalamus, this is manifested as increased rs-FC with the superior parietal lobule, inferior frontal gyrus, lingual gyrus, and pre-cuneus, and decreased connectivity with inferior parietal lobule, supplementary motor area, and anterior cingulate gyrus. And contradictory results were noted in the insula and middle frontal gyrus (Amin et al., 2018; Qin Z. X. et al., 2020; Figure 9).

It is noteworthy that many studies have analyzed differences between migraine with aura (MA) and migraine without aura (MwoA). First, 192 rs-FCs were predominantly located in the occipital lobe, sensorimotor network, part of the medial-cerebellum, cingulate-orbitofrontal network, DMN, and FPN, capturing the features of MwoA and identified as neural biomarkers (Tu et al., 2020). Compared to HC, MwoA exhibited reduced rs-FC homogeneity (rs-FCHo) in the insula, limbic regions (cingulate cortex), and thalamus, with decreased FC between insula and anterior cingulate cortex, pontine nuclei to superior parietal lobule, middle temporal gyrus and middle frontal gyrus, as well as between the anterodorsal nucleus and the left precuneus, ventroposterior nucleus and the pre-cuneus, inferior parietal lobule and right middle frontal gyrus, while ReHo presented contradictory results (Zhang J. et al., 2016; Lo Buono et al., 2017; Qin Z. et al., 2020; Qin Z. X. et al., 2020). Similarly, spontaneous ALFF reductions in the spinal trigeminal nucleus and hypothalamus were observed in MwoA during the pre-ictal phase (Kim Y. E. et al., 2021). Both during and between MA attacks, increased FC from the left pontine nuclei to the primary somatosensory cortex and increased sub-low frequency oscillatory power were detected (Hougaard et al., 2017; Meylakh et al., 2018). The occipital cortex and the extracorporeal visual cortex geniculate, particularly the lingual gyrus, were also found to play a crucial role in the ictal and interictal phases of MA (Russo et al., 2019b). In patients with complex MA, the rs-FC in the left lingual gyrus within the visual network and the right anterior insula within the sensorimotor network were significantly higher compared to simple migraine and MwoA (Silvestro et al., 2022). Changes in rs-FC during the period between migraine attacks in patients MA and MwoA were also observed in the visual cortex and a diverse array of brain regions involved in visual processing (including the middle frontal area, insula, anterior cingulate gyrus, superior parietal lobule, and cerebellum), although there are still discrepancies between studies (Arca et al., 2021; Ashina et al., 2021).

Some differentiated studies have also been undertaken on patients with two different courses, chronic migraine (CM) and episodic headache. The hypothalamic cluster exhibits a heightened BOLD signal response to painful stimulation of the trigeminal nerve in CMs relative to episodic migraine and HC (Schulte et al., 2017). Using bilateral caudate nuclei as seeds, we discovered that the FC values between the right caudate nucleus and brain regions primarily involving emotion, cognition, and sensory processing were elevated in CM patients compared to HCs and those with episodic migraines (Yuan et al., 2022). Additionally, increased rs-FC was observed between the spinal trigeminal nucleus and hypothalamus in CM patients, whereas no such phenomenon was observed in episodic migraine patients (Lerebours et al., 2019). Similarly, hippocampal rs-FCD increased in CM patients, while decreased in MwoA (Dai et al., 2021). Other research has shown that, in CM, the connectivity between the executive control network and the default mode network as well as the dorsal attention system is reduced, while the interconnectivity between the latter two is increased, which may be related to the severity of headaches (Coppola et al., 2019).

#### 2.2.2. Efficacy of machine learning and advanced statistical modeling based on fMRI data in migraine diagnosis

**FIGURE 10:**
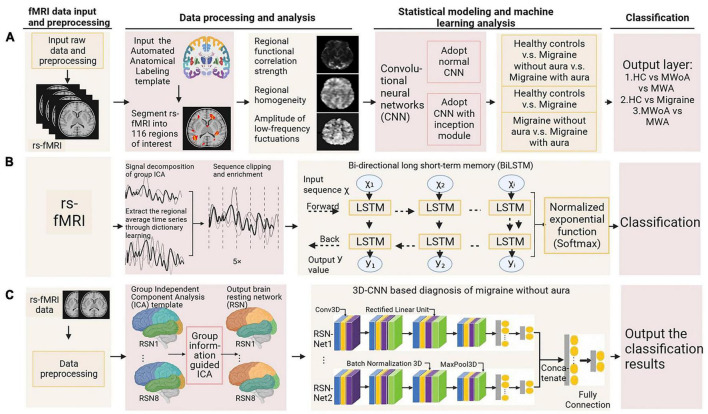
Machine learning and advanced statistical modeling of fMRI in migraine diagnosis. Adapted from **(A)** Yang et al. (2018), **(B)** Sun et al. (2023), and **(C)** Li et al. (2022b), respectively.

In the context of migraine research, Yang et al. (2018) extracted three features (ALFF, ReHo, and strength of regional FC) from rs-fMRI data of migraine patients, mapped these to functional images, and applied a deep learning approach based on the Inception module of CNN. The CNN, utilizing these three rs-fMRI features, was capable not only of distinguishing migraine patients from HC, but also separating two subtypes of migraine. This method achieved an accuracy rate exceeding 90% for classifying migraine and HC, as well as MwoA and MA. Specifically, under the feature of strength in regional FC, the Inception-based CNN achieved the highest recognition rate of 99.25% with an area under the curve (AUC) of 0.99 when distinguishing between the HC and the migraine group (Figure 10A). Sun et al. (2023) utilized group-level ICA and dictionary learning to segment regions of interest (ROIs) in the brain. Based on the ROIs, they extracted regional average time series signals, segmented and expanded them into subsequences, and trained a model within a bidirectional long short-term memory (BiLSTM) network. Capturing the dependency of neuronal activity over time, the softmax function was used for binary classification. Migraine patients and HCs achieved a classification accuracy of 96.94%, with an AUC of 0.98, and a relatively short computation time (Figure 10B). Nie et al. (2023) extract reliable features based on recursive feature elimination of support vector machines, and obtain the optimal subset of features by eliminating suboptimal features one by one while maximizing the accuracy of feature association classification. An automated identification framework for migraine patients and HC was developed, using rs-FC intensity signatures and dynamic FC patterns (DFCPs). Their findings suggest that the classification effect of DFCP attributes is superior to rs-FC strength characteristics. However, when the time window length is 24 s, combining these two features results in the best performance, with the best accuracy of 96.81% and the best precision of 95.41%. Xiao et al. (2018) used linear-SVM, k-nearest neighbor, Radial Basis Function SVM (RBF-SVM) and decision tree to classify and compare migraine and HC respectively. The results showed that in the classifier trained with fine features, the linear -SVM and RBF-SVM both obtained the best classification accuracy rate of 93.97%. Jorge-Hernandez et al. (2014) developed a set of machine learning algorithms through graph theory analysis, demonstrating that the classification accuracies of migraine patients using NN and SVM classifiers were 92.86% and 87%, respectively. Wang Q. et al. (2022) combined degree centrality (DC) and SVM analysis, revealing a significant decrease in the DC values of the bilateral inferior temporal gyrus (ITG) among migraine sufferers. A positive linear correlation was found between the left ITG and MIDAS scores. SVM results indicated that the DC value of the left ITG has the potential to serve as an imaging diagnostic biomarker for migraines, with utmost diagnostic precision, sensitivity, and specificity rates of 81.82, 85.71, and 77.78%, respectively. The above results show that machine learning on fMRI data has excellent discrimination ability in the differentiation between migraine and HC. Compared with other models, NNs and SVMs have better discrimination ability, especially after fine feature training, or combined with some other feature data, can obtain better diagnostic accuracy.

Furthermore, there have been studies investigating the diagnostic efficacy of migraine subtypes. Li et al. (2022b) have proposed an intelligent auxiliary diagnostic algorithm for MwoA diagnosis, using a 3 dimensions CNN model. Compared to HC, this method achieved a maximum diagnostic accuracy of 98.40% (Figure 10C). Zhang Q. et al. (2016) utilized rs-fMRI data to extract three features and combine them with regional gray matter volume data from structural MRI. They employed a multi-kernel strategy for feature selection and combination, followed by training an SVM classifier to differentiate subjects at the individual level. A leave-one-out cross-validation method was employed to evaluate the performance of the classifier, the final classification accuracy for distinguishing MwoA patients and HCs was 83.67% (sensitivity 92.86%, specificity 71.43%), where the anterior cingulate cortex, prefrontal cortex, orbitofrontal cortex, and insular cortex contributed the most discriminative features. Fu et al. (2022) utilize fALFF per voxel and use sophisticated machine learning of fMRI to confirm abnormal fALFF patterns and extract them as features for constructing discriminant models by SVM. They demonstrated that the trigeminal neck complex/ventral medial medulla (TCC/RVM), cingulate mesocortex (MCC), medial prefrontal cortex (mPFC), and temporoparietal junction are key brain regions to distinguish migraine. High accuracy (79.3%), sensitivity (78.6%), and specificity (80.0%) were demonstrated in differentiating MwoA patients from HC. When further identification of patients with chronic migraine, the ability of machine learning to distinguish is equally significant. Chong et al. (2017) utilized machine learning techniques to analyze the FC of 33 pain-related seed regions in individual rs-fMRI data, finding that the rs-FC of the right middle temporal lobe, posterior insula, mid-cingulate cortex, left ventromedial prefrontal cortex, and bilateral amygdala regions best differentiated migraine patients from HC. Forward stepwise search using diagonal quadratic discriminant analysis, as well as an in-house developed machine learning pipeline (encoded in MATLAB), to determine which principal components contribute to classification accuracy. A 10-fold cross-validation revealed an optimal classification accuracy of 86.1%, with higher accuracy of 96.7% in patients with extended illness duration (>14 years) and 82.1% in those experiencing shorter duration (≤14 years). Chen et al. (2019) found that, compared to HCs, both episodic migraine and CM patients had significantly reduced hypothalamic volumes as observed through high-resolution fMRI. This offered good diagnostic accuracy for CM (sensitivity 81.25%, specificity 100%). Morphometric analysis of voxels indicated a positive linkage between diminished anterior hypothalamic volume and the incidence of headaches in CMs, whereas a negative correlation was found with the reduced volume of the posteromedial hypothalamus and the frequency of both episodic migraines and CMs. Studies have also been conducted to distinguish migraine from other confusing secondary headaches. Dumkrieger et al. (2022) included clinical features, structure, and functional resting state measurements as latent variables. Fit the data using a classifier for principal component ridge logistic regression. Calculate the average accuracy using the Leave One cross-validation. Through internal variable selection and principal component creation, the average accuracy of FC data was 72%, and the average accuracy of FC-free data was 63.4%.

In summary, machine learning and statistical modeling using commonly employed fMRI data have demonstrated good diagnostic performance for migraine. Furthermore, these methods indicate that patients with longer disease duration may have higher classification accuracy. However, the diagnostic performance is slightly lower when differentiating migraine subtypes such as those without aura or with aura, and comparing chronic or episodic migraine with HC. Further in-depth studies involving similar machine learning and statistical modeling approaches can potentially distinguish migraine subtypes more effectively and improve classification accuracy.

### 2.3. Diagnosis of TTH based on fMRI technology

#### 2.3.1. Exploring the pathophysiology of TTH diagnosis through fMRI technology

Tension-type headache is characterized by dull, constricting, or pressing band-like pain around the head and is the most common but under-researched primary headache disorder with an incompletely understood pathophysiology. The initial whole-brain voxel-wise fALFF research performed utilizing rs-fMRI data in a study, and the results showed increased fALFF in the right posterior and anterior insula in episodic TTH sufferers compared to HC, decreased fALFF in the posterior cingulate cortex, and a negative correlation between right anterior insula fALFF and TTH attack frequency (Yang et al., 2023). Further research detected lower ALFF values in TTH across six frequency bands, predominantly localized to the middle and superior frontal gyri (Li et al., 2021). ReHo analysis of the spontaneous neural activity in TTH across various frequency bands demonstrated augmented ReHo within the right medial superior frontal gyrus in the conventional band (0.01–0.08 Hz) and slow-5 band (0.01–0.027 Hz), when compared to HC (Zhang S. et al., 2021). However, a previous study found significantly reduced ReHo values in the bilateral caudate nucleus, subcallosal area, shell nucleus, left middle and superior frontal gyrus in the TTH group (Wang et al., 2014). These contradictory results may arise from disparities in diagnostic criteria and severity of TTH across studies.

#### 2.3.2. Efficacy of machine learning and advanced statistical modeling based on fMRI data in TTH diagnosis

Wang Y. et al. (2022) selected the bilateral amygdalae and left hippocampi as seed regions for rs-FC analysis, assessing their discriminative capacity for TTH sufferers compared to HC and migraineurs. According to the rs-FC between the left amygdala and bilateral corpus callosum/cuneus, the AUC, sensitivity, and specificity for discriminating migraines from TTH were 0.822, 78.3, and 81.8%. Based on the rs-FC between the left amygdala and left Heschl’s gyrus, the AUC, sensitivity, and specificity for discriminating migraines from TTH were 0.868, 82.6, and 81.8%. Using the rs-FC between the left amygdala and right Heschl’s gyrus, the AUC, sensitivity, and specificity for discriminating migraines from TTH were 0.830, 78.3, and 86.4%. Due to the restricted number of studies and their sample size, the aforementioned findings must be treated with discretion until further validation of the research’s reproducibility is achieved.

### 2.4. Diagnosis of TAC based on fMRI technology

#### 2.4.1. Exploring the pathophysiology of TAC diagnosis through fMRI technology

**FIGURE 11:**
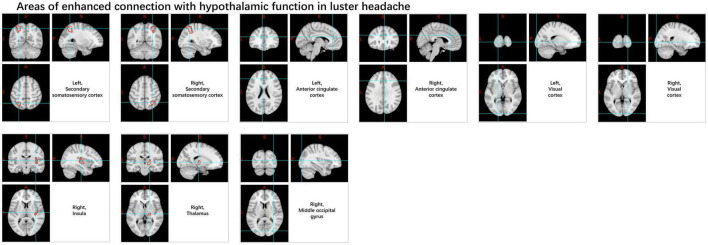
Changes in the functional connectivity of the hypothalamus in patients with cluster headache.

Trigeminal autonomic cephalalgia encompasses a collection of distinct primary headaches, characterized by moderate or severe one-sided pain located in the distribution area of the first branch of the trigeminal nerve, and is combined with cranial autonomic symptoms and signs. The low occurrence rate of TAC presents a challenge in carrying out large-scale investigations and randomized clinical trials (Diener et al., 2023). The prevailing theory for years has been that the hypothalamus triggers, and then activates the trigeminal autonomic reflex, a hypothesis many accept to explain chronic cluster headache, which is the most frequently observed condition in this category (Hoffmann and May, 2018). FMRI analysis mainly focuses on FC and abnormal activation in cortical areas, brainstem, and hypothalamus (Sprenger et al., 2004; Matharu and Zrinzo, 2010; Morelli et al., 2013; Chiapparini et al., 2015; Naegel and Obermann, 2021). ICA and seed correlation analysis of rs-fMRI data showed increased FC between the hypothalamus and the pre-genual anterior cingulate cortex, visual cortex, bilateral secondary somatosensory cortex, right middle occipital gyrus, right thalamus, and right insula in cluster headache patients compared to HC (Figure 11). And variations within the sensorimotor and primary visual networks in CH patients displayed a reduction (Rocca et al., 2010). Another study indicated a strong reduction in FC in the right frontal pole and right amygdala pathways in chronic cluster headache patients compared to HC (Ferraro et al., 2022). Rs-fMRI analysis revealed that, compared to the control group, left-sided cluster headache patients displayed decreased fALFF in the left cerebellum, left putamen, left frontal lobe, left anterior cingulate, and right posterior central gyrus, while right-sided cluster headache patients exhibited decreased fALFF in the right cerebellum, right cingulate gyrus, right superior parietal lobule, right inferior parietal lobule, right posterior central gyrus, and left precuneus, with no areas showing increased fALFF (Chen Y. et al., 2022). Another rarer subtype is short-lasting unilateral neuralgiform headache attacks with conjunctival injection and tearing (SUNCT); several fMRI studies (May et al., 1999; Matharu et al., 2003; Auer et al., 2009) conducted during multiple attack periods showed activation of the posterior hypothalamus, brainstem, right pre-central, superior frontal, inferior frontal, middle frontal cortex, and bilateral supplementary exercise area.

#### 2.4.2. Efficacy of machine learning and advanced statistical modeling based on fMRI data in TAC diagnosis

Messina et al. (2023) collected clinical, functional, and structural MRI data from migraineurs, cluster headache sufferers, and HCs. By employing ICA and SVM algorithms for classification analysis, this supervised machine learning approach, in combination with multimodal imaging data, resulted in an 89% classification accuracy for differentiating migraines from HCs and a 98% accuracy for differentiating cluster headaches from HCs. The accuracy in distinguishing between cluster headaches and migraine patients reached 99% when using a combined MRI-clinical classification model. Bilateral hypothalamic and PAG functional networks were the most important fMRI features for classifying migraine and cluster headache patients from the control group. Comparison revealed that, unlike migraine patients, those with cluster headaches showed diminished functional interactions between the left thalamus and cortical areas, areas crucial for interoception and sensory integration, serving as the most discriminative fMRI feature for distinguishing cluster headache patients.

### 2.5. Diagnosis of other primary headaches based on fMRI technology

Other primary headaches include those induced by coughing, exertion, or sexual activity. Research has analyzed a subtype of headache within this category known as new daily persistent headache (NDPH) (Robbins et al., 2010), characterized by the sudden onset of daily headaches lasting for 3 months or longer, with secondary causes excluded. A study (Qiu et al., 2023) using structural magnetic resonance imaging combined with multimodal brain imaging analysis of magnetoencephalography found that NDPH patients had abnormal brain morphology such as cortical area, cortical thickness, and gray matter volume, accompanied by abnormal cortical neural activity. Another study (Wang et al., 2023) using fMRI to map abnormal FC in NDPH patients showed abnormal FC in multiple brain regions involved in emotion and pain perception and regulation, but these abnormalities did not correlate with clinical features. All in all, the exploration appears to be only superficial. Studies on other primary headaches are scarce and may require further classification; therefore, this review cannot provide a summary of research related to diagnosis in this category.

## 3. Current status and future prospects

With the development of science and technology, many technologies such as biomarker research (Singhal et al., 2023), neuroimaging technology (Aberathne et al., 2023; Castaldo et al., 2023), artificial intelligence and machine learning (Rahaman et al., 2020, 2021; Chen A. et al., 2022; Inoue et al., 2023), and remote monitoring and sensor technology (Lu et al., 2023; Rahman et al., 2023) have been widely used to explore various diseases and achieve interesting results. Machine learning discussed in this article has been the hottest topic in the field of artificial intelligence in the past 10 years, among which deep learning, classification, neural networks, etc. are the most common keywords in this field (Nam et al., 2022; De la Vega Hernández et al., 2023). Similarly, fMRI studies have gradually developed a complete system from focused activity and functional connections to complex networks and dynamic models. In particular, research on psychiatric disorders, such as the large-scale collaborative project that has been carried out and integrated multicenter fMRI datasets from ten research institutions and clinical hospitals in China, reveals the reproducible disruption of functional connectomes in patients with major depressive disorder, providing a promising paradigm for understanding pathology and exploring clinical biomarkers (Xia et al., 2022; Zhang et al., 2023).

Although in terms of signal acquisition, BOLD-fMRI technology is an indirect measurement of neural activity, which limits the temporal resolution, and various unpredictable factors may interact with the signal and reduce its signal-to-noise ratio, resulting in BOLD signal loss or spatial distortion. But continuous technological improvements are addressing these shortcomings (Glover, 2011; Campbell and Weber, 2022). Bibliometric analysis shows that, especially after 2017, research on fMRI combined with machine learning has grown considerably, enabling deeper exploration of neuronal characteristics behind individual behaviors and brain diseases, and imaging biomarkers for prediction have gradually been identified (Sun et al., 2021). However, the use of these emerging technologies in primary headaches is still minimal. This may be due to the fact that many studies of the pathophysiology of primary headache have been conducted in recent years, but its pathogenesis is still poorly understood, and it has not been well connected with new technologies (Ferrari et al., 2022).

Therefore, fMRI combined with machine learning is still an area of active research in the diagnosis of primary headache, whether to develop biomarkers or classification algorithms, or to use fMRI data alone or in combination with structural MRI data (Zhang Q. et al., 2016; Tu et al., 2020). This trend may be due to the lack of attention paid to diseases such as TTH, which is also associated with lower rates of medical visits due to milder symptoms. There is also considerable heterogeneity in the assessment of headache disorders based on ICHD because of the inevitable subjectivity (Kopel and Gottschalk, 2022). In addition, it is difficult to obtain a large sample size when conducting clinical research, the selection process may have different criteria, and the image changes at different stages of the disease may be different, which makes it difficult to carry out multicenter research data sharing. There are currently no publicly available datasets for fMRI for primary headache, and the studies included in our review are all single-center clinical studies with various heterogeneities in the study process, resulting in low reproducibility of design and results. Best practices for improving reproducibility include method transparency, eliminating errors, using prior assumptions and power calculations, using standardized instruments and diagnostic criteria, and developing large-scale, publicly available datasets (Hranilovich et al., 2023). In the future, on the basis of the pathophysiology obtained by fMRI exploration, more statistical models and deep learning models should be combined, or other technical means such as blood biomarkers and structural magnetic resonance. For example, more large-scale, multi-center, large-sample studies are carried out to realize the transformation of individual diagnosis from the concept stage to the clinical application stage.

## 4. Limitations

There are some limitations to this article. First, there is a lack of consistent objective indicators to assess headache severity and frequency of attacks, so this section is less summarized here. Second, further classification of each primary headache subtype is considered as there are few and no studies that are primarily discussed herein. Third, because the results of the current fMRI exploration of the pathophysiology of primary headache have not been standardized, all studies in machine learning analysis have collected and created datasets independently. Last but not least, statistical modeling and machine learning based on fMRI data for the diagnosis of primary headache is still scarce, and this article only summarizes the characteristics of each study in detail, and has not yet been able to further unify the results of all studies.

## 5. Conclusion

This article describes the latest classification and epidemiology of primary headache, summarizes the principles and implementation methods of fMRI technology, and reviews the latest progress in data analysis and diagnostic model design of primary headache and its subtypes based on fMRI technology. It covers classical analytical methods for studying pathophysiology and combines them with advanced statistical models and machine learning. FMRI studies associated with primary headaches reveal functional changes in different brain regions, and machine learning and statistical modeling have demonstrated their excellent diagnostic performance, especially when combined with other data such as structural MRI. In addition, patients with a longer course of disease may also increase diagnostic accuracy. However, the number of studies is limited and reproducible is low, so the results need to be interpreted carefully. The continuous improvement of fMRI technology and machine learning models, and the in-depth study of primary headache provide great potential for diagnosis and prediction.

With the advent of artificial intelligence and big data in healthcare, these advances are expected to contribute to precision medicine for functional neurological disorders such as primary headaches. This review provides a bridge for medical and engineering researchers who are committed to combining advanced imaging technology and artificial intelligence technology for neurological disease research, provides powerful tools and ideas, and also provides a reference for clinicians or neurology researchers, helps to understand the pathophysiological mechanism and diagnostic methods of primary headache in a deeper way, and encourages us to do more exploration in the diagnosis of primary headache.

## Author contributions

M-LL: Conceptualization, Data curation, Methodology, Software, Visualization, Writing—original draft, Writing—review and editing. FZ: Visualization, Writing—original draft, Writing—review and editing. Y-YC: Writing—original draft, Writing—review and editing. H-YL: Writing—original draft, Writing—review and editing. Z-WQ: Writing—review and editing. Y-FW: Writing—review and editing. L-TH: Methodology, Supervision, Validation, Visualization, Writing—original draft, Writing—review and editing. J-HW: Funding acquisition, Writing—original draft, Writing—review and editing.
