# Supplementary material for: A state-of-the-art review of functional magnetic resonance imaging technique integrated with advanced statistical modeling and machine learning for primary headache diagnosis
Source: Front Hum Neurosci. 2023 Sep 1;17:1256415. doi: 10.3389/fnhum.2023.1256415 (PMC10513061; doi:10.3389/fnhum.2023.1256415)
Supplement: Supplementary file 1 [file Table_1.docx]

**Table S1.** The main method of rs-fMRI data analysis.

| **Category** | **Items** | **Definition** |
| --- | --- | --- |
| Functional segregation | Amplitude of low-frequency fluctuations (ALFF) analysis | ALFF values correlate with the strength of local neural activity and are commonly used to measure BOLD signals in the frequency range of 0.01 to 0.1 Hz |
|  | Regional homogeneity (ReHo) analysis | ReHo describes the synchronicity of a voxel with a time series of neighboring voxels. The higher the ReHo value, the better the consistency between local voxels and neighboring voxels, but it does not necessarily indicate more significant local neural activity. |
| Functional integration | Functional connectivity density (FCD) analysis | FCD shows the functional connection strength of a voxel by calculating the correlation of the BOLD time series between a voxel and other voxels, rather than the specific connection path. |
|  | Seed-based functional connectivity analysis | Calculate the BOLD time series correlation coefficient between a subpoint and all other voxels or regions of interest (ROI) in the brain to derive the functional connections between brain regions. Seed points or ROIs can be based on a priori assumptions or other literature results, or they can be based on ALFF, ReHo, or FCD results. |
|  | Independent component analysis (ICA) | Using the blind source separation method, the spatially independent and time-series related functional networks are separated, and several resting brain networks are usually separated, including but not limited to: default network, auditory network, accent network, executive control network, visual network, sensorimotor network, dorsal visual network (frontal parietal attention network), etc. |
|  | Graph theory-based brain network analysis. | Graph theory has been widely used to study the properties of complex networks, describing the efficient and orderly information transfer of the brain locally and as a whole, and providing a theoretical framework for analyzing the topology of brain networks. |
