# Supplementary material for: A state-of-the-art review of functional magnetic resonance imaging technique integrated with advanced statistical modeling and machine learning for primary headache diagnosis
Source: Front Hum Neurosci. 2023 Sep 1;17:1256415. doi: 10.3389/fnhum.2023.1256415 (PMC10513061; doi:10.3389/fnhum.2023.1256415)
Supplement: Supplementary file 2 [file Table_2.docx]

**Table S2.** Application range, advantages and disadvantages of commonly used models for machine learning.

| Model | Application range | Advantages | Disadvantages |
| --- | --- | --- | --- |
| Linear Regression | It is used to model the linear relationship between continuous target variables and features. Suitable for prediction and regression problems. | Easy to use, has high computational efficiency, and has a good effect on linear relationship modeling. | Only applies to linear relationships, does not perform well for nonlinear relationships. |
| Logistic Regression | It is used to build a classification model and classify samples by logical functions. Suitable for binary or multivariate classification problems. | Suitable for binary classification problems, and has good probability modeling and strong interpretability. | Limited ability to model nonlinear relationships and feature interactions. |
| Decision Trees | Build tree models based on feature decision rules that can be used for classification and regression problems. Have intuitive explainability and an easy-to-understand decision-making process. | Easy to interpret, can handle numeric and categorical features, and can capture nonlinear relationships and feature interactions. | Easy to overfit, unstable, sensitive to noise and data changes. |
| Random Forests | An ensemble model of multiple decision trees that improves accuracy and robustness by voting or averaging predictions. Suitable for classification and regression problems. | The overfitting problem of decision trees is improved, and it has good accuracy and robustness. | The model is complex, and the computational overhead for large-scale data and high-dimensional data is large. |
| Support Vector Machines | By finding the optimal hyperplane to achieve binary classification, the kernel function is supported to extend to nonlinear classification. Suitable for binary or multivariate classification problems. | It can effectively model complex relationships in high-dimensional space and has good generalization performance. | Sensitive to large-scale data and noise, and sensitive to parameter selection and kernel function selection. |
| K-Nearest Neighbors | Based on the distance metric between features, classification or regression is carried out by selecting the closest K-neighbors. Suitable for classification and regression problems. | Easy to use, good for modeling local patterns, and is suitable for nonlinear problems. | The calculation overhead is large, it is sensitive to outliers, and it is difficult to process data with sample imbalance and high dimension. |
| Neural Networks | Computational models that simulate neuronal networks in the human brain for complex nonlinear classification and regression problems. | Able to approximate arbitrarily complex functions, perform well on large-scale datasets, and adapt to nonlinear problems. | The computational complexity is high, sensitive to parameter initialization and selection, and architecture design, and requires a large amount of data for training. |
| Deep Learning | Deep learning is an extension of neural networks. The technology automatically creates multi-level models with hierarchical representations of input data and allows for powerful automated feature extraction. Deep learning models are being used more frequently to improve diagnostic capabilities and discover patterns in patient data that characterize diseases. | With strong expression ability, suitable for complex data processing such as images and speech, it can reach or exceed the human level in many tasks. | It requires a large amount of data and computing resources, and model training and tuning are relatively difficult and poorly interpretable. |
| Naive Bayes | Based on Bayes' theorem and the assumption of feature conditional independence, rapid classification is realized. Suitable for problems such as text classification and spam filtering. | Simple and fast, effective for working with high-dimensional data and large-scale datasets, robust for missing data. | Sensitive to correlations between features, assuming that features are independent of each other. |
