# Supplementary material for: A state-of-the-art review of functional magnetic resonance imaging technique integrated with advanced statistical modeling and machine learning for primary headache diagnosis
Source: Front Hum Neurosci. 2023 Sep 1;17:1256415. doi: 10.3389/fnhum.2023.1256415 (PMC10513061; doi:10.3389/fnhum.2023.1256415)
Supplement: Supplementary file 3 [file Table_3.docx]

**Table S3.** Specific characteristics of included studies combining machine learning with fMRI for primary headache diagnosis.

| Author (Year) | Participant characteristics and number | Datasets acquisition | Data feature extraction and machine learning model selection | Model training and validation | Optimal diagnostic accuracy | Results and discussion |
| --- | --- | --- | --- | --- | --- | --- |
| Yang et al. (2018)([Yang et al., 2018](#_ENREF_13)) | 21 patients with migraine without aura (MwoA), 15 patients with migraine with aura (MA), and 28 healthy controls (HCs). The diagnosis of migraine is made by a neurologist according to the criteria of the International Classification of Headache Disorders, Second Edition (ICHD-II). | The rs-fMRI data were collected using planar echo image sequences and analyzed using statistical parameter mapping software and resting fMRI data processing assistants. Perform slice timing correction, realignment, motion correction, spatial normalization, bandpass filtering and other preprocessing for the time series of each voxel. Finally, the low-frequency fluctuation amplitude, regional uniformity, and regional function-related intensity are calculated. | Deep learning methods based on the Inception module with GoogleNet and convolutional neural networks based on the AlexNet module, combined with data based on the three functional measurements of rs-fMRI (low-frequency fluctuation amplitude (ALFF), regional uniformity, and regional functionally related intensity). | During training, about 80% of the subjects in the three groups (51 people in total) were assigned to the training dataset, and the remaining 20% (13 people in total) were used for testing purposes. The neuroimaging package Nibabel loads the preprocessed rs-fMRI time series data into memory and then decomposes it into a 2D (x, y) matrix along the z and time (t) axes. Use Python and OpenCV to convert 2D matrices to lossless PNG format. Then, the deep learning platform Tensorfow is used to classify the data and converted to h5py format, and the SoftMax function is selected as the output layer classifier. | The best accuracy was 99.25% for a deep learning method based on a convolutional neural network with the Inception module of GoogleNet combined with regional functionally correlated strength to diagnose migraine and healthy participants. | Convolutional neural network (CNN) improves classification performance compared to traditional support vector machines (SVM). regional functional correlation  The regional functional correlation strength (RFCS), regional homogeneity (ReHo) and ALFF can be used to represent different degrees of classification features. Among them, RFCS can improve the accuracy and obtain the best classification results. Limitations are the small sample size and lack of models that integrate relevant signal data with specific brain regions. |
| Sun et al. (2023)([Sun et al., 2023](#_ENREF_9)) | 64 adults participated in rs-fMRI data acquisition, including 36 migraine patients, as a group of migraine patients. Migraine patients were rated by neurologists according to ICHD-II. 28 people as HCs. | The rs-fMRI data were acquired with gradient echo-planar imaging sequences. The scanning time of all subjects was 6min (ie 360s), corresponding to 180 time points. Then remove the first 10 time points of each functional data, leaving 170 time points (ie 340s). Temporal layer correction and realignment were then performed, and the resulting images were registered to a standard space of 3 mm voxels using the Montreal Neurological Research template. Finally, each voxel time series was band-pass filtered (0.01–0.08 Hz). | For small sample populations, group-level independent component analysis (ICA) and dictionary learning were used to divide different brain regions, and then the regional average time series signals were extracted. Second, the extracted time series is evenly divided into sub-time series to augment the model input sample. | Further study of the before and after time series information within each time series to characterize periodic brain state changes. The BiLSTM network is used on the basis of rs-fMRI time series clipping expansion. At the same time, 8-fold cross-verification was carried out to calculate sensitivity, specificity and classification accuracy. | Using GroupICA combined with DictLearning to extract features, combined with BiLSTM with 32 hidden nodes, the accuracy rate reached the highest 96.94%. | Compared with the traditional model, the model proposed in this paper fully considers the time-varying characteristics and the prediction efficiency of the model, and can be fast  Efficiently capture brain dynamics associated with characteristic states from rs-fMRI data. The limitation lies in the small sample size and data set based on the deep learning framework. It also did not combine the properties of BOLD signaling to characterize changes in brain function in migraine patients. |
| Nie et al. (2023)([Nie et al., 2023](#_ENREF_8)) | 34 patients with migraine were diagnosed according to ICHD-III. The rs-fMRI data of 34 normal control subjects were obtained from a free public database published by the Biomedical Research Center of Excellence. | In the migraine group, the number of scanning slices = 38 (covering all brain regions), the repetition time (TR) = 3.0 s, and the number of time points = 160. In the control group, the number of scanned slices = 33 (covering all brain regions), TR = 2.0 s, and the number of time points = 140. Data preprocessing was performed by slice timing, head motion correction, spatial normalization, and spatial smoothing. | The static functional connection (sFC) of each subject was estimated from the time series using the Pearson correlation method, the dynamic functional connection (dFC) was extracted by sliding time window, and the window lengths of 12 s, 24 s, 36 s, 48 s and 60 s were selected in steps of 1 to explore the effect of different window lengths on performance. The dFCS matrix was divided and several divisions were extracted; For each fragment, a whole-brain quasi-stable connectome pattern vector is obtained based on time averaging. | Recursive feature elimination (SVM−RFE) based on SVM extracts reliable features, and the optimal feature subset is obtained by eliminating suboptimal features one by one under the condition of maximizing the accuracy of feature association classification. Run the SVM−RFE method once per cross-validation fold. A linear SVM classifier is then used to evaluate classification performance. | On the 24 s time window, the sFC intensity feature and the dynamic functional connectivity pattern (DFCP) feature obtained the best accuracy of 96.81%, and the best precision of 95.41%. | Limitations: Analytical barriers due to data heterogeneity between different data sources. There is no use of the adaptive variable point method to identify the length of the time window, which would make it difficult to generalize. There are insufficient data to further distinguish between the various subtypes of migraine, the severity of migraine, and the different primary headaches. |
| Xiao et al. (2018)([Xiao et al., 2018](#_ENREF_12)) | 17 clinically confirmed migraine patients were used as the experimental group and 100 normal people as healthy control groups. | The rs-fMRI data were collected using single-excitation-sensitive gradient-echo plane imaging. Statistical Parametric Mapping is used to preprocess the data, mainly for time layer correction, head movement correction, image standardization, etc. The autodissection label template divides the brain into 90 brain regions (excluding cerebellar regions). | Autoencoders automatically obtain the features of the data to be analyzed. Design the input layer, output layer, the number of neurons and the activation function model of each hidden layer, and perform iterative training for 7000 times to become a fully trained deep autoencoder. According to the features obtained by the automatic dissection template, as the original features, the features automatically obtained by the autoencoder, as the fine features, respectively use these two features to compare the accuracy achieved by various classification algorithms. Use linear-SVM and k-nearest neighbor respectively, radial basis function SVM (RBF-SVM) and decision tree for classification. | Select 10 original features of migraine and 10 original features of healthy people as training data, and train 4 kinds of classifiers and deep autoencoders. The remaining 7 migraine data and the data of 7 healthy control groups randomly selected were used as test data to verify the correct classification rate of various classifiers. | Among the classifiers trained by fine features, linear-SVM and RBF-SVM both have the best classification accuracy of 93.97%. | The features extracted by deep learning can effectively improve the classification accuracy rate in the classification results of various classifiers. Further research can obtain more migraine data and use more sample data to train the classifier, thereby improving the classification accuracy of the classifier. The classification effect provides an important image reference for clinical diagnosis of migraine. |
| Jorge-Hernandez et al. (2014)([Jorge-Hernandez et al., 2014](#_ENREF_5)) | 15 healthy participants, 20 sporadic migraine participants, and 19 participants with migraine and substance abuse. | All subjects underwent an fMRI examination lasting approximately 8 minutes. T1 images with high-resolution reconstructed patient anatomy were used as sequences for morphological evaluation, consisting of 250 layers. Ultrasound plane imaging blood oxygen level dependent (EPI-bold) was used as the functional sequence, with a total of 214 dynamically measured sequences. Brain atlas are used to understand the brain regions analyzed. And preprocess the data with some motion correction tools, etc. | Using graph theory research algorithms from Clustering, Path Length, and Dispersion, categorical features are extracted. Subjects are then classified using machine learning algorithms based on a combination of supervised classifiers (linear discriminant analysis (LDA), SVM, neural networks (NN), k-nearest neighbors), unsupervised classifiers (K-means), and semi-supervised classifiers (AdaBoost). | Ten subjects were selected from one group, 10 HCs as exercises, and the rest as validation. | The NN classifier received the best classification accuracy of 92.86% and SVM was 87.18%. | The limitation is that the sample size is small. It is still not possible to combine an autoclassifier with a full atlasis or for personal use. |
| Wang et al. (2022a)([Wang et al., 2022a](#_ENREF_10)) | Twenty-seven patients with a neurology diagnosis of migraine based on ICHD-II were included in the study. 28 healthy subjects, as HC. | The rs-fMRI scan totaled 36 layers and scanned the whole brain for 8 minutes. A T1-weighted fast scrambled phase gradient echo sequence was used to acquire structural images and scan 176 layers. The whole brain scan time is 4.2 minutes. Preprocessing of static data is performed as follows: time layer correction, head motion correction, spatial normalization, linear drift, regression to remove covariates (head motion parameters, white matter signal, cerebrospinal fluid signal) and filtering. | The degree centrality (DC) is used to calculate the Z-value DC distribution plot. The DC values of migraine patients and control patients were analyzed by two separate sample t-tests, and DC altered brain regions were identified. | The SVM was conducted using a library for SVM (LIBSVM, RRID: SCR_010243) software package in Matlab (RRID: SCR_001622). The LIBSVM classifier is trained to learn differences between groups by providing examples in form of (xi, ci), where x represents the DC values of abnormal clusters and c standards for the class label. The grid search method and Gaussian radial basis function kernels were used for parameter optimization. The “leave-pair-out” cross-validation approach was applied using the LIBSVM software to achieve the highest sensitivity and specificity. | Performing SVM analysis to determine DC values in bilateral inferior temporal gyrus can be used to differentiate between patients and HC. The DC value in the left inferior temporal gyrus achieved the best accuracy of 81.82%. | A normal DC value of the left ITG can be used for the clinical diagnosis of migraine. Limitations are small sample sizes and most patients are already treated with migraine medications, which may have affected the results. and failure to distinguish between migraine subtypes. |
| Li et al. (2022)([Li et al., 2022](#_ENREF_6)) | 60 MwoA, 65 HCs matched the sex and age of the MwoA. All patients were diagnosed by a neurologist according to ICHD-III criteria. | The rs-fMRI data were acquired using echo plane imaging sequences. Then preprocessing such as time correction, head motion correction, spatial standardization, and spatial smoothing are carried out. | Based on the new 3D-CNN technology designed, a MwoA intelligent auxiliary diagnosis algorithm, MwoA3D-Net, is proposed, which adopts the ICA method guided by group information to generate resting-state brain networks (RSN). | The RSN generation module is used to generate RSNs related to MwoA, and then the obtained RSNs are used to train the MwoA3D-Net algorithm. Eight RSN-Net optimal models are loaded, all RSNs of the participants are used as input, forward propagation is performed and the loss is calculated, and finally the fully connected layer of the MwoA3D-Net algorithm is trained by backpropagation. The data is verified based on the 5-fold cross-validation method. All data are randomly and evenly divided into 5 copies, 1 of which is taken each time for the test set, and the remaining 4 data for the training set. At the same time, this paper randomly divides the 4 training sets into 5 parts, 1 of which is used as the verification set, and the remaining 4 as the training set. Finally, the ratio of training set, verification set, and test set is 16:4:5 for each fold of data. | The diagnostic accuracy of MwoA3D-Net for MwoA reached 98.4%. | The diagnostic accuracy of MwoA3D-Net is higher than that of SVM, AlexNet, CNN with Inception, MB-CNN, and 3D ResNet. The limitation is that the sample size is small, but a series of optimization strategies are adopted for the problem of overfitting of small samples. In addition, the MwoA3D-Net algorithm has achieved good results, but there are still problems such as a large number of model parameters and the lack of capture of instantaneous functional changes in the extracted functional connection features. |
| Zhang et al. (2016)([Zhang et al., 2016](#_ENREF_14)) | 21 MwoA patients and twenty-eight HCs participated in the study. The diagnosis of MwoA is diagnosed by a neurologist based on ICHD-II. | Acquisition of T1-weighted structural images using fast gradient echo sequences prepared by 3D magnetization and rs-fMRI data using planar echo imaging (EPI) sequences. The structural images were preprocessed using the statistical parametric mapping software SPM8. Preprocess resting functional images using the SPM8 and the resting fMRI Data Processing Assistant toolbox. The time series of each voxel was filtered with bandpass (0.01 Hz-0.08 Hz), and finally ALFF, ReHo and RFCs were calculated. Structural MRI data were simultaneously obtained to measure regional gray matter volume. | Partition the whole brain using an automated anatomical labeling atlas to define regions of interest. For each measurement, the values ​​of 116 predefined regions of interest are extracted as classification features. Features are first selected and combined by a multi-kernel strategy; then an SVM classifier is trained to distinguish subjects. | The performance of the classifier is evaluated by the left-one cross-validation method. One sample is designated as a test sample, and the remaining samples are used to train the classifier. | The best classification accuracy obtained by this classifier under the most discriminating features contributed by the anterior cingular cortex, prefrontal cortex, orbitofrontal cortex and insular lobe was 83.67%. | Using ALFF, ReHo, RFCS, and GM features derived from rs-fMRI and structural MRI scans, and with multicore SVM integration, classification performance is improved. The limitations are the small sample size and lack of a large database, making the classifier specific only to the current dataset. People with MwoA had higher depressive anxiety scores and may have biased the data. |
| Fu et al. (2016)([Fu et al., 2022](#_ENREF_4)) | 70 MwoA diagnosed with ICHD-II and 70 HCs participated in the study. | An fMRI scan was performed to acquire T1-weighted structural images and rs-fMRI image data. Preprocessing was performed in the Brain Imaging Data Processing and Analysis 3.0 tool (DPABI 3.0), fractional amplitudes of low-frequency fluctuations (fALFF) calculations per voxel were performed, and the differences between HC and MWoA were compared. | Meaningful voxels are extracted as features for constructing discriminant models from SVM. | Use left-one cross-validation to avoid the risk of overfitting. Use a linear SVM algorithm combined with F-Score's feature selection to get the best classifier. | Discriminatory features were found in the trigeminal cervical complex/rostral ventromedial medulla (TCC/RVM), thalamus, medial prefrontal cortex (mPFC), and temporal gyrus, with the best identification accuracy for a biomarker containing 3,650 features 79.3%. | It was confirmed that abnormal fALFF patterns have the potential to become neuroimaging biomarkers with high accuracy in MWoA classification. A limitation is the lack of comparisons of different subtypes of migraine. Only the SVM algorithm was adjusted, and the differences of other algorithms were not compared. This was a single-centre study that had not been externally validated. |
| Chong et al. (2017)([Chong et al., 2017](#_ENREF_2)) | There were 108 participants (58 migraine patients and 50 HCs). All migraine patients were diagnosed with paroxysmal or chronic migraine (CM) according to ICHD-II. HC is made up of community members who have never had migraines. | Scan and collect high-resolution T1 and T2 weighted imaging data for all participants for 10 min each. Perform pre-processing such as slice time correction, motion correction, and realignment with the first volume, skull and non-brain tissue resection, spatial smoothing, and functional scanning. | Apply seed-based (region of interest (ROI)) correlation analysis and calculate the Fisher r-z transformation map by extracting the time course of each seed region (33 seed regions in total) and calculating the correlation of each seed region with each voxel throughout the brain. | Based on the ROI, 66,347 data points are generated using principal component analysis (PCA) to create a linear combination of data points based on the original voxels, called the principal component, PC. These PC "sets" are then used to build classification models. Forward stepwise search using diagonal quadratic discriminant analysis, as well as an in-house developed machine learning pipeline (encoded in MATLAB), to determine which PCs contribute to classification accuracy. Finally, a 10-fold cross-validation method is used to evaluate classification accuracy. | The best accuracy for classifying individual migraine patients with HC based on the rs-fc pattern of only six ROIs (right middle temporal lobe, right posterior insulin, right middle cingulate gyrus, bilateral amygdala, and left ventromedial prefrontal lobe) was 86.1%. And relatively speaking, patients with migraine with a longer course (> 14 years) were more accurately classified, reaching 96.7%. | The brain's resting state functional connectivity model can accurately classify about 8 out of 10 migraine patients from healthy controls. Functional neuroimaging defines migraine patients with a longer course more accurately than migraine patients with a shorter duration, suggesting that burden of disease may drive functional restructuring. A limitation is that the sample size is small and does not allow classification of migraine subtypes. |
| Chen et al. (2015)([Chen et al., 2019](#_ENREF_1)) | 18 MwoA, 16 CMs and 21 HCs. All patients were ICHD-III compliant. | High resolution structural images were acquired with a three-dimensional T1-weighted fast spoiled gradient recalled echo (3D T1-FSPGR) sequence. Resting-state functional MR images were obtained using a gradient echo-planar imaging (EPI) sequence. One hundred and eighty axial EPI functional volumes  were obtained over 6 min. Oblique axial T2-weighted imaging (T2WI), T1 fluid-attenuated inversion recovery (T1-FLAIR) and diffusion weighted imaging (DWI) were also acquired. | The volume of HTH was calculated and voxel-based morphometry (VBM) was performed over the whole HTH. | Receiver operating characteristics (ROC) curve analysis was applied to evaluate the diagnostic efficacy of HTH volume. Correlation analyses with clinical variables were performed and FC maps were generated for positive HTH regions according to VBM comparison. | The critical volume of the hypothalamus was 1.429 ml and had good diagnostic accuracy for CM with a sensitivity of 81.25% and a specificity of 100%. | Structural plasticity and FC alterations in HTH occur in interictal EM and CM. HTH volume < 1.429 ml may have good diagnostic value for CM and be considered a biomarker for CM. Limitations include significant age and sex differences between groups. Differences in hypothalamic volume between headache patients and those without headache were not compared. Migraine-related neuropeptides released by the hypothalamus in the blood were not measured. |
| Dumkrieger et al. (2022)([Dumkrieger et al., 2022](#_ENREF_3)) | There were 34 migraine patients (30 CM and 4 paroxysmal migraines) and 48 patients with persistent post-traumatic headache. The patient is diagnosed by a headache specialist based on ICHD-III. | 3D T1-weighted sagittal Magnetization Prepared Rapid Gradient Echo, axial T2-weighted imaging, Diffusion Tensor Imaging (DTI) (nonlinear directions and one image without diffusion weighting) and ten minutes of blood oxygenation level dependent (BOLD) resting state imagine data were collected. | Clinical data, structural and fiber tract measures were standardized, converted to principal components and a logistic ridge regression model was fit on the principal components (PC). | Leave one out cross-validation was used to assess model performance and the ridge L2 regularization parameter was set within the cross-validation loop. Within each cross validation loop an additional, inner, leave one out cross validation (81-fold) was performed for each candidate regularization parameter. The candidate ridge parameter with the best performance over the inner cross validation loops was chosen as the ridge parameter for the primary, outer, cross validation loop. The addition of the many (4692) functional connectivity derived variables necessitated variable selection prior to model fitting. | With internal variable selection and principal components creation, FC data were 72% accurate in distinguishing persistent post-traumatic headache from migraine. | Multivariate models based on clinical characteristics, fc, and brain structural data accurately classify and differentiate PPTH vs. migraine suggesting differences in the neuromechanism and clinical features underlying both headache disorders. |
| Wang et al. (2022b)([Wang et al., 2022b](#_ENREF_11)) | There were 24 HCs, 24 migraine patients, and 24 tension headache (TTH) patients. The ages, sex ratios and years of schooling matched in the three groups. The patient is diagnosed by two neurologists based on ICHD-III. | Resting fMRI data were collected by echo plane imaging. Image preprocessing using DPARSF software. | Bilateral amygdala and hippocampus were selected as seed regions for resting functional connectivity (rsFC) analysis to explore potential rsFC features that may distinguish between the two types of headache. Calculate the average time series for each seed in each subject. Then, the Pearson correlation coefficient between the average time course of seeds and the average time history of each voxel in the whole brain is calculated. Apply the Fisher z-transform to improve the normality of the correlation coefficients. Finally, rsFC plots for each seed for each subject are obtained. | Whole-brain voxel analysis of rsFC betwel differences for each seed using one-way covariance analysis (ANCOVA), and post-hoc multiple comparisons of clusters showing significant differences in one-way ANCOVA using Tukey's test to test pairwise differences between groups (HC vs. migraine, HC vs. TTH, and migraine vs. TTH). The accuracy of potential rsFC alterations for differentiating migraine patients from non-migraine patients was evaluated using ROC analysis. | Based on the rs-FC between the left amygdala and left Heschl's gyrus, the AUC, sensitivity, and specificity for discriminating migraines from TTH were 0.868, 82.6%, and 81.8%. | The amygdala and hippocampus with rsFC of the occipital lobe can be used to distinguish between migraine sufferers and people with TTH. Limitations are other primary migraines that are not further classified. Pre-specified seed points can cause other seed points that may have excellent diagnostic effects to be missed. The study sample size was small. |
| Messina et al. (2023)([Messina et al., 2023](#_ENREF_7)) | Twenty patients with migraine, 20 patients with cluster headache, and 15 HCs were included. All patients met the diagnostic criteria for paroxysmal migraine and paroxysmal cluster headache in the International Classification of Headache Disorders. | By fluid-attenuated inversion recovery, 3-dimensional (3D) T1-weighted gradient echo, diffusion-weighted (DW) spin-echo, resting state  (RS) functional MRI (fMRI) and 3D pseudocontinuous arterial spin labeling to acquire brain sequences. Assess the presence of white matter (WM) high signal on FLAIR and measure the volume using local threshold segmentation techniques. The data were preprocessed and analyzed to obtain data from brain volume, WM score anisotropy (FA), WM mean diffusivity (MD), cerebral blood flow (CBF), and RS functional connectivity (FC) plots. | The linear nuclear SVM model and step-by-step removal process are used to assess the most accurate classification of patients and controls. | Each feature is ranked in relative importance when classifying patients and controls, as well as patient subgroups, based on the weight vectors provided by the model. After each round of SVM training, the least informative metric is removed and a new SVM is trained with the remaining metrics. Repeat this process until only one feature remains. Record the accuracy of the classifier at each phase removal. During cross-validation, the dataset is divided into n parts. The model is then trained n-1 times and its performance is validated on the remaining times. | The classification accuracy was 89% for migraine from control and 98% for cluster headache and control. The MRI-clinical data combined classification model is 99% accurate in differentiating between patients with cluster headache and migraine. | MRI classifiers include brain function and structural MRI measurements that accurately classify individuals as migraine or cluster headaches. Patients with migraine and cluster headache share functional biomarkers, including the hypothalamus and periductal gray matter network. A limitation is that participants with both migraine and cluster headache may confuse important points of distinction. Analysis of episodic and interictal periods was not available. |

**References：**

Chen, Z., Chen, X., Liu, M., Ma, L., and Yu, S. (2019). Volume of Hypothalamus as a Diagnostic Biomarker of Chronic Migraine. *Front Neurol* 10**,** 606. doi: 10.3389/fneur.2019.00606.

Chong, C.D., Gaw, N., Fu, Y., Li, J., Wu, T., and Schwedt, T.J. (2017). Migraine classification using magnetic resonance imaging resting-state functional connectivity data. *Cephalalgia* 37(9)**,** 828-844. doi: 10.1177/0333102416652091.

Dumkrieger, G., Chong, C.D., Ross, K., Berisha, V., and Schwedt, T.J. (2022). The value of brain MRI functional connectivity data in a machine learning classifier for distinguishing migraine from persistent post-traumatic headache. *Front Pain Res (Lausanne)* 3**,** 1012831. doi: 10.3389/fpain.2022.1012831.

Fu, C., Zhang, Y., Ye, Y., Hou, X., Wen, Z., Yan, Z., et al. (2022). Predicting response to tVNS in patients with migraine using functional MRI: A voxels-based machine learning analysis. *Front Neurosci* 16**,** 937453. doi: 10.3389/fnins.2022.937453.

Jorge-Hernandez, F., Garcia Chimeno, Y., Garcia-Zapirain, B., Cabrera Zubizarreta, A., Gomez Beldarrain, M.A., and Fernandez-Ruanova, B. (2014). Graph theory for feature extraction and classification: a migraine pathology case study. *Biomed Mater Eng* 24(6)**,** 2979-2986. doi: 10.3233/bme-141118.

Li, X., Wei, B., Wu, H., Li, X., Hong, Y., and Cong, J. (2022). 3D-CNN auxiliary diagnosis algorithm for migraine without aura. *Computer Engineering and Applications* 58(04)**,** 169-176.

Messina, R., Sudre, C.H., Wei, D.Y., Filippi, M., Ourselin, S., and Goadsby, P.J. (2023). Biomarkers of Migraine and Cluster Headache: Differences and Similarities. *Ann Neurol* 93(4)**,** 729-742. doi: 10.1002/ana.26583.

Nie, W., Zeng, W., Yang, J., Zhao, L., and Shi, Y. (2023). Classification of Migraine Using Static Functional Connectivity Strength and Dynamic Functional Connectome Patterns: A Resting-State fMRI Study. *Brain Sci* 13(4). doi: 10.3390/brainsci13040596.

Sun, A., Chen, N., He, L., and Zhang, J. (2023). [Research on migraine time-series features classification based on small-sample functional magnetic resonance imaging data]. *Sheng Wu Yi Xue Gong Cheng Xue Za Zhi* 40(1)**,** 110-117. doi: 10.7507/1001-5515.202206060.

Wang, Q., Gao, Y., Zhang, Y., Wang, X., Li, X., Lin, H., et al. (2022a). Decreased degree centrality values as a potential neuroimaging biomarker for migraine: A resting-state functional magnetic resonance imaging study and support vector machine analysis. *Front Neurol* 13**,** 1105592. doi: 10.3389/fneur.2022.1105592.

Wang, Y., Wang, Y., Bu, L., Wang, S., Xie, X., Lin, F., et al. (2022b). Functional Connectivity Features of Resting-State Functional Magnetic Resonance Imaging May Distinguish Migraine From Tension-Type Headache. *Front Neurosci* 16**,** 851111. doi: 10.3389/fnins.2022.851111.

Xiao, J.C., Zeng, W.M., Yang, J.J., Shi, Y.H., Xu, Y.H., and Jiao, L. (2018). fMRI Data Analysis Based on Deep Learning in the Application of Migraine. *Computer system applications* 27(04)**,** 249-253. doi: 10.15888/j.cnki.csa.006344.

Yang, H., Zhang, J., Liu, Q., and Wang, Y. (2018). Multimodal MRI-based classification of migraine: using deep learning convolutional neural network. *Biomed Eng Online* 17(1)**,** 138. doi: 10.1186/s12938-018-0587-0.

Zhang, Q., Wu, Q., Zhang, J., He, L., Huang, J., Zhang, J., et al. (2016). Discriminative Analysis of Migraine without Aura: Using Functional and Structural MRI with a Multi-Feature Classification Approach. *PLoS One* 11(9)**,** e0163875. doi: 10.1371/journal.pone.0163875.
